# Investigation of Heat Transfer Enhancement in a Triple Tube Latent Heat Storage System Using Circular Fins with Inline and Staggered Arrangements

**DOI:** 10.3390/nano11102647

**Published:** 2021-10-09

**Authors:** Xinguo Sun, Hayder I. Mohammed, Mohammadreza Ebrahimnataj Tiji, Jasim M. Mahdi, Hasan Sh. Majdi, Zixiong Wang, Pouyan Talebizadehsardari, Wahiba Yaïci

**Affiliations:** 1 Jiangsu Smart Factory Engineering Research Centre, College of Management and Engineering, Huaiyin Institute of Technology, Huai’an 223003, China; 2 Department of Physics, College of Education, University of Garmian, Kurdistan, Kalar 46021, Iraq; hayder.i.mohammad@garmian.edu.krd; 3 Department of Mechanical Engineering, Qom University of Technology, Qom 1519-37195, Iran; ebrahimnataj.m@qut.ac.ir; 4 Department of Energy Engineering, University of Baghdad, Baghdad 10071, Iraq; jasim@siu.edu; 5 Department of Chemical Engineering and Petroleum Industries, Al-Mustaqbal University College, Hillah 51001, Iraq; hasanshker1@gmail.com; 6 China Water Resources Pearl River Planning Surveying & Designing Co., Ltd., Guangzhou 510610, China; xiongwz2020@126.com; 7 Centre for Sustainable Energy Use in Food Chains, Institute of Energy Futures, Brunel University London, Kingston Lane, Uxbridge UB8 3PH, UK; 8 CanmetENERGY Research Centre, Natural Resources Canada, 1 Haanel Drive, Ottawa, ON K1A 1M1, Canada

**Keywords:** staggered and inline fins, fins’ dimensions, phase-change material, thermal energy storage, melting

## Abstract

Inherent fluctuations in the availability of energy from renewables, particularly solar, remain a substantial impediment to their widespread deployment worldwide. Employing phase-change materials (PCMs) as media, saving energy for later consumption, offers a promising solution for overcoming the problem. However, the heat conductivities of most PCMs are limited, which severely limits the energy storage potential of these materials. This study suggests employing circular fins with staggered distribution to achieve improved thermal response rates of PCM in a vertical triple-tube heat exchanger involving two opposite flow streams of the heat-transfer fluid (HTF). Since heat diffusion is not the same at various portions of the PCM unit, different fin configurations, fin dimensions and HTF flow boundary conditions were explored using computational studies of melting in the PCM triple-tube system. Staggered configuration of fin distribution resulted in significant increases in the rates of PCM melting. The results indicate that the melting rate and heat charging rate could be increased by 37.2 and 59.1%, respectively, in the case of staggered distribution. Furthermore, the use of lengthy fins with smaller thickness in the vertical direction of the storage unit resulted in a better positive role of natural convection; thus, faster melting rates were achieved. With fin dimensions of 0.666 mm × 15 mm, the melting rate was found to be increased by 23.6%, when compared to the base case of 2 mm × 5 mm. Finally, it was confirmed that the values of the Reynolds number and inlet temperatures of the HTF had a significant impact on melting time savings when circular fins of staggered distribution were included.

## 1. Introduction

It is widely accepted, nowadays, that the key strategy for transforming the global energy system into a green and sustainable economy is to accelerate the uptake of renewable energy sources, such as solar- and wind-based sources, across all types of energy use [1,2]. Such energy sources make it possible to address the consequences of harsh fossil-fuel usage such as climate change and environmental pollution, which highly impact the quality of human life [3,4]. However, the inherent fluctuations in the availability of energy from these sources necessitate the development and incorporation of effective storage systems to render possible their stable operation and continuous power generation [5,6]. An efficient method for storing energy that is widely acknowledged in the field of renewable energy utilization is its conversion into thermal energy [7,8]. This method is known as thermal energy storage (TES) [9,10]. It can provide energy storage on a daily, weekly, monthly, or even seasonal basis, allowing for any potential mismatch between energy supply and demand to be addressed [11,12]. As a result, there is increasing interest in improving the design of TES systems to be able to provide remarkable energy savings with a significant impact on a wide range of renewable energy applications [13,14].

TES is basically classified as sensible, latent, or thermochemical, depending on how heat is held by the storage material. The latent TES, which employs so-called phase-change materials (PCMs), is preferred above the others. The reasons for this are twofold. First, PCM-based heat storage systems are much more compact as compared to sensible heat technologies such as rock or water tanks. The volume ratio of PCM-based TES to water tank or rock-bed TES is about 1 to 8 or 1 to 17, respectively [15]. This feature critically assists the portability and mobility of the TES system when the volume/mass of the storage material is a design limitation. Second, the thermophysical properties of phase transition in PCMs allow for minimal or no temperature stratification; thus, the temperature during operation can remain almost unchanged. Therefore, PCMs find significant applications in the fields of energy savings in buildings [16,17,18,19], load management in district cooling utilities [20,21] and peak shaving in plants of renewable energy [22,23,24].

The useful application of PCMs as TES materials largely depends on their thermophysical properties, such as melting/solidifying point, specific heat capacity, latent enthalpy of fusion, thermal diffusivity and heat conductivity. In recent years, there has been increasing interest in modifying these properties in a way that allows for all the difficulties and inconsistencies linked to this TES technology to be addressed. One main difficulty that most present-day PCMs suffer from is their poor heat conductivity, which has a major impact on the PCM responding rates to the energy charge/discharge functions [25]. Due to this, the PCMs’ thermal responsiveness should be improved so that the stored heat of fusion can be released or recovered at a pace that is suitable for the desired application. From a design perspective, the selection of adequate PCM casing and the incorporation of the proper mass/volume of the enhancement material are two crucial steps for overcoming issues of low PCM-to-demand responding rates. The high conductivity nanoparticles [26,27], extended fins [28,29,30,31,32,33,34] and porous matrices [35,36,37,38,39] are among the most famous enhancement materials. Applying fins to increase the heat-transfer area in charge is considered the most traditional technique for improving the thermal response in energy systems. The high enhancement ratio, minimal manufacturing costs and ease of installation are only a few benefits when fins are successfully applied [40]. Therefore, studies are becoming increasingly interested in optimizing the shape, sizing and material usage of fins to intensify the energy charging/discharging performance in PCM-based TES systems.

The circular fin is considered the easiest fin design to fabricate and install, thus, it is of distinct interest to this present study [40]. Lacroix [41] reported that melting improvement of PCM due to the introduction of circular fins was more influencing at lower heat-transfer fluid temperatures and flow rates. Jung and Boo [42] suggested applying greater fin pitch to enhance the thermal reaction rate of PCM, particularly during the discharging mode. Yang et al. [43] succeeded to save the melting time by 65% when circular fins were included in the shell-and-tube system. For obtaining a good role of natural convection during melting, Singh et al. [44] recommended a non-uniform distribution with a gradual decrease in the circular fin height, so that up to 43% charging-time saving could be achieved. However, Shahsavar et al. [45] reported that homogeneous fin distribution could save melting time by around 24 percent compared to the corresponding non-uniform fin distribution. Yang et al. [46] found that applying fins with irregular distributions can reduce the average temperature of PCMs by about 34% and save 63% on melting time.

As stated above, the latent TES poses a number of disputes, including the selection of the proper casing design to facilitate good heat transmission between the circulating HTF and the PCM implemented. In this context, TTHEs (triple-tube heat exchangers) have received a lot of attention in recent years [47]. They consist of three concentric tubes, the center of which is filled with a PCM, while the two outside tubes are filled with HTF. Since both walls of the annulus housing the PCM can be thermally active, a larger heat exchange area than in a typical double-pipe heat exchanger can be preserved between the PCM and the HTF. This allows for faster thermal response rates on the PCM side to be achieved [48]. This study mainly aimed to examine the possible enhancement on melting of PCMs in a novel TTHE arrangement with circular fins, where the HTF flows in one direction (gravity direction), while it flows oppositely in the other direction. This arrangement would promote a better role of natural convection during the PCM charging mode. Another aim is to analyze the thermal performance of the, by connecting five circular fins to each of the tubes, as well as their efficacy, by optimizing their size and location under different thermal and flow conditions of the HTF. Emphasis is also given to comparing the thermal response of TTHE with applying two forms of fin distribution between the inner and outer tubes, namely, the inline and staggered fin distribution, based on the overall energy and the charging rate produced by each case.

## 2. System Description

**Figure 1:**
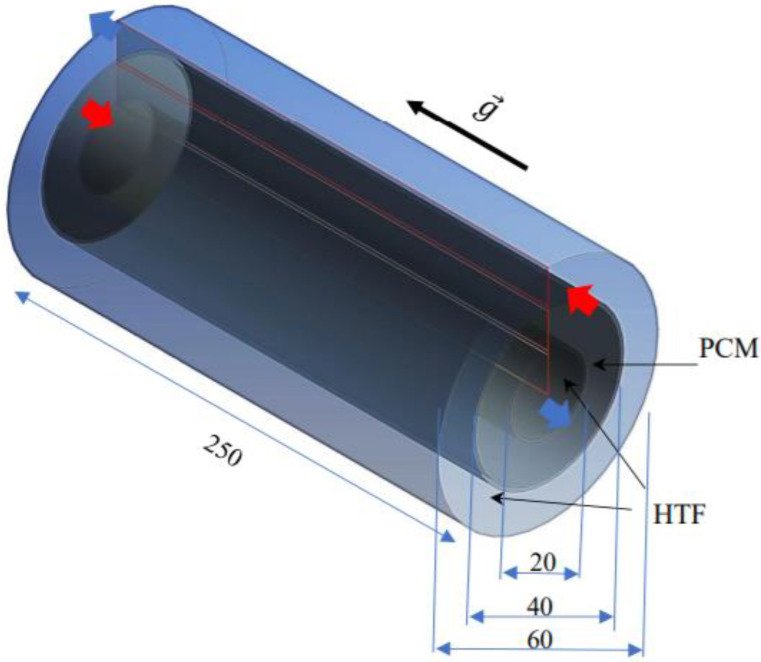
The schematic of the studied triple-tube heat exchanger (all the dimensions are in mm).

The heat exchanger investigated in this study was a triple-pipe heat exchanger with and without considering circular fins in the PCM domain. The fins were considered in the two arrangements of inline and staggered fins. The proposed system without fins is shown in Figure 1. The length of the system was 250 mm, while the inner, middle and outer diameters of the pipes were 20, 40 and 60 mm, respectively. The thickness of the tube was also considered 1 mm. The PCM filled the middle pipe, while water, as the heat transfer fluid (HTF), passed through the inner and outer pipes to release heat to the PCM. The HTF passed opposite to the gravity direction in the inner pipe, while it passed in the gravity direction in the outer tube. Thus, the flow of the HTF in the heat exchanger was counter-current, as recommended in the literature. For the inlet boundary, velocity and temperature of the HTF were known, while outflow conditions were considered for the HTF outlet. For the first case (studying the effects of the sizes and distribution of the fins), the velocity was considered as Re = 1000 and the inlet temperature was 50 °C. For the case of investigating the effect of the velocity, the Re was considered as 500, 1000 and 1500, at 50 °C. For the investigation of the effect of the inlet temperature, three different values of the inlet temperature were used (45, 50, 55 °C). The external wall of the system was assumed to be insulated.

**Figure 2:**
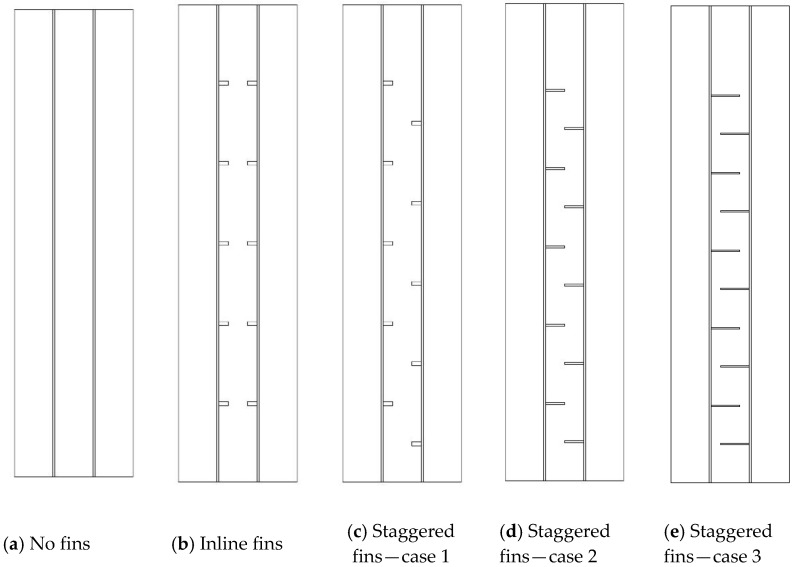
Various configurations of fins studied in this paper: (**a**) no-fins, (**b**) inline fins, (**c**) staggered fins, case 1, (**d**) staggered fins, case 2, and (**e**) staggered fins, case 3.

Due to the nature of the studied problem and lack of circumferential variation in the flow, the system was considered axisymmetric; as shown in Figure 2, which is displayed in an axisymmetric situation. The axisymmetric domain is also shown in Figure 1 with red lines. The fins were connected to the inner and middle pipes to increase the heat transfer rate to the PCM. As shown, five fins were connected (total number of fins is 10) in the forms of inline (Figure 2b) and staggered (Figure 2c–e). In the staggered arrangement of the fins, three different dimensions were studied, considering a similar volume for the fins to have a meaningful comparison and to have a similar PCM mass in all the studied systems. Note that, for the system without fins, the length of the pipe was determined to have a similar PCM mass compared with the finned cases. The dimensions of the fins were 2 mm in thickness and 5 mm in length for the systems with inline fins (Figure 2b) and case 1 in the staggered demonstration (Figure 2c). The length of the fins for the systems with a staggered arrangement of the fins was 10 mm in Figure 2d and 15 mm in Figure 2e. Note that the thickness of the fins was then calculated to have the same volume for the fins. Note that the distances between the fins were considered 40 mm and, in the staggered arrangement, the first fin was placed at 20 mm from the bottom of the heat exchanger.

The main criteria for selecting the dimensions of the fins in the finned geometries was considering a similar area of the fins for all the studied cases. In other words, the effect of different arrangements of inline and staggered were studied to enhance the performance of the system, considering equal volume for the fins in the domain. It is true that, by increasing the volume of the fins in the domain, higher heat transfer can be achieved; however, on the other hand, a higher volume for the PCM domain is also required to have a constant PCM mass. Thus, the aim of this study is to find the best performance of the system considering a constant volume for the fins and thus constant mass for the PCM in a specific volume.

The inlet temperature and Reynolds number for the HTF were 50 °C and 1000, respectively, to find the best configuration for the fins. In addition, as a sensitivity analysis, the inlet temperature and Reynolds number of the HTF were also studied for the best case. The initial temperature of the PCM considered was 15 °C.

## 3. Mathematical Modeling

For the simulation of the phase change process, the enthalpy–porosity method, introduced by Brent et al. [37,49], was employed, with the porosity and the liquid fraction considered similar in each cell in the computational domain. The Boussinesq approximation for density variation of the PCM was considered to determine the natural convection effect in the domain due to the variation in the density of the PCM. The fluid flow was laminar and transient and was also considered incompressible and Newtonian. The viscous dissipation was also neglected and the no-slip boundary condition was used for the walls [45,50]. The adiabatic boundary condition was considered for the outer wall of the heat exchanger and for PCM walls at the bottom and top of the heat exchanger, which is meaningful, considering a high-quality insulation. With these assumptions, the conservation equations of continuity, momentum and energy are then given as [51]

(1) $$\frac{\partial \rho }{\partial t}+\nabla .\rho \vec{V}=0$$

(2) $$\rho \frac{\partial \vec{V}}{\partial t}+\rho \left(\vec{V}.\nabla \right)\vec{V}=-\nabla P+\mu \left(\nabla^{2}\vec{V}\right)-\rho_{ref}\beta \left(T-T_{ref}\right)\vec{g}-\vec{S}$$

(3) $$\frac{\rho C_{p}\partial T}{\partial t}+\nabla \left(\rho C_{p}\vec{V}T\right)=\nabla \left(k\nabla T\right)-S_{L}$$

The last term in the momentum equation ($\vec{S})$ is added due to the effect of the phase change process, which is the damping term of Darcy’s law, defined as [52]

(4) $$\vec{S}=A_{m}\frac{{\left(1-\lambda \right)}^{2}}{\lambda^{3}+0.001}\vec{V}$$

where the mushy zone constant $A_{m}$ is considered 105, according to the literature [23,53,54]. For the effect of latent heat and phase-change process in the energy equation, a source term is added, where λ (liquid fraction of PCM) is introduced as [55]

(5) λ=ΔHLf={0      if  T<TSolidus1      if  T>TSolidusT−TSolidusTLiquidus−TSolidus      if  TSolidus<T<TLiquidus}

The source term $S_{L}$ in the energy equation is obtained as follows:

(6) $$S_{L}=\frac{\rho \partial \lambda L_{f}}{\partial t}+\rho \nabla \left(\vec{V}\lambda L_{f}\right)$$

The rate of stored energy during the melting process is then defined as

(7) $$\dot{E_{T}}=\frac{E_{e}-E_{i}}{t_{m}}$$

where $t_{m}$ is the melting time and $E_{e}$ and $E_{i}$ are the total energy of the PCM at the end and beginning of the melting process. E is the summation of sensible heat $\left(MC_{p}dT\right)$ and latent heat $\left(ML_{f}\right)$ of the PCM.

It should be noted that the governing equations for the HTF flow are the conventional Navier–Stokes equations, where the effect of phase change is eliminated from Equations (1)–(3). The HTF flow is laminar, in this study.

## 4. Numerical Modeling, Grid Independence and Validation

To solve the governing equations of the heat exchanger and phase change process, the ANSYS-FLUENT software was used by applying the SIMPLE algorithm for the pressure–velocity coupling and the Green–Gauss cell-based method for computing the variables’ gradients. Moreover, the QUICK differencing scheme was used to solve the momentum and energy equations, while pressure correction equations were used by adopting the PRESTO scheme. The under-relaxation factors were considered to be 0.3, 0.3, 0.5 and 1 for pressure correction, velocity components, liquid fraction and energy equation, respectively. The convergence criteria for the continuity, momentum and energy equations were set to be 10^−4^, 10^−4^ and 10^−6^, respectively.

**Figure 3:**
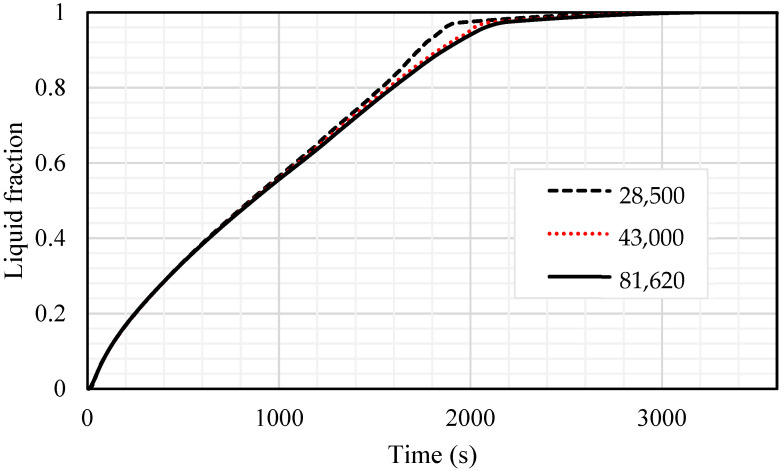
The variation of liquid fraction for different sizes of the gird.

Before starting the simulations, the grid independence analysis, as well as time step size analysis, were investigated. For the mesh independency analysis, different sizes of the mesh, 28,500, 43,000 and 81,620, for the number of cells were evaluated using the time step size of 0.2 s for the inline-finned case with uniform fin arrangement. The results are displayed in Figure 3. These results are almost identical for the grid sizes of 43,000 and 81,620; therefore, the mesh size of 43,000 was chosen for further analysis. It should be noted that the different sizes of 0.1, 0.2 and 0.4 s for the time step size for the selected grid were also investigated and 0.2 s was selected for the size of the time step.

**Figure 4:**
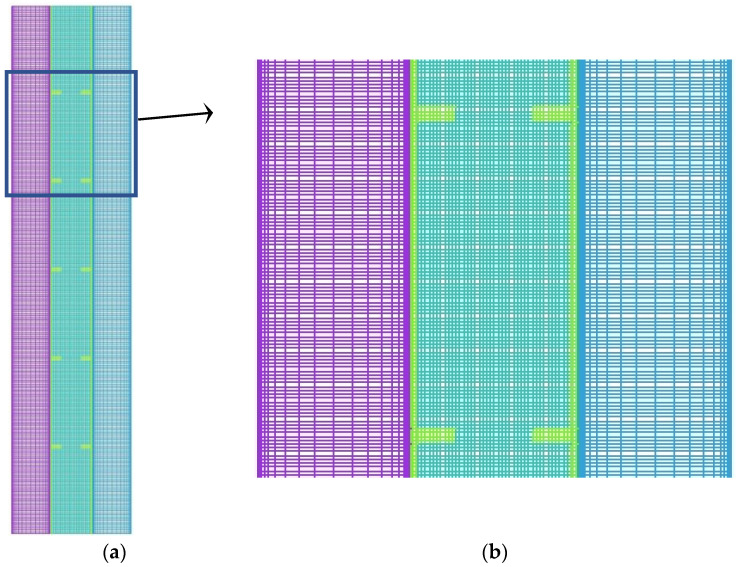
The configuration of the mesh after grid independence analysis (using 43,000 cells): (**a**) entire domain, (**b**) section zoom.

The schematic of the grid selected after grid independent test with 43,000 cells is illustrated in Figure 4.

**Figure 5:**
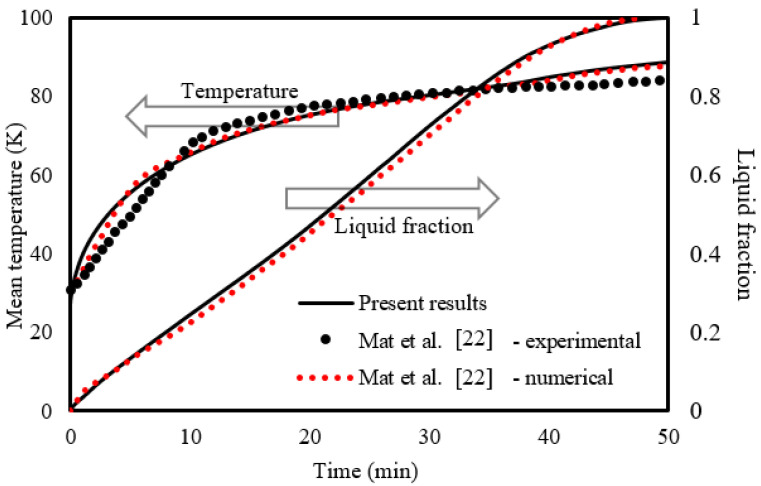
Verification of the numerical model.

To validate the code, the experimental and numerical results of Mat et al. [23] were employed. In the study of Mat et al., the effect of fins attached to both outer and inner surfaces of the PCM zone (RT58) in a triple-tube TES unit was experimentally studied. The unit was made up of three concentric copper tubes 500 mm long. The outer and middle tubes featured 2 mm thick walls, while the inner tube had a 1.2 mm thick wall. The middle tube was used to house the PCM, while the other two tubes were utilized to circulate water as the HTF. In the validation, the same initial and boundary conditions from the cited study were used in the present study. A comparison between the two studies is illustrated in Figure 5. As seen, the presented results are in line with the experimental data for the temperature and numerical data for the melt fraction of Mat et al. [23]. Over the whole simulation, the highest relative error (% e = |(Ω_Exp_ − Ω_Num_)|/Ω_Exp_) reported was 2%. Here, Ω is the simulation dependent variable (temperature or liquid fraction); Exp and Num are indices representing the referential experimental findings and the present numerical predictions, respectively.

## 5. Results and Discussion

This section explores the impacts of the fins’ distribution pattern and the size on the melting process. The staggered fin pattern is compared with the uniform (inline) mode and the no-fin cases. The best case was used to evaluate the influence of the inlet temperature and the velocity (represented in Reynold number) of the heat transfer fluid (water) on the melting behavior. Those parameters were evaluated by analyzing the contour images and the development figures of liquid fraction and temperature.

### 5.1. Effect of Fin Addition in the Forms of Inline and Staggered Compared with the No-Fin Case

Utilizing fins into the TES increases the melting rate due to enlarging the surface area of the heat transfer and enhancing the average thermal conductivity for the entire domain, as the thermal conductivity of the metal fins is higher than that of the PCM. Adding fins helps deliver heat to the deep region in the PCM domain, enhancing the thermal distribution. Further, once the PCM melts, the convective heat transfer is affected more by the presence of fins. This part of the study included five fins with a length of 5 mm and a thickness of 2 mm.

**Figure 6:**
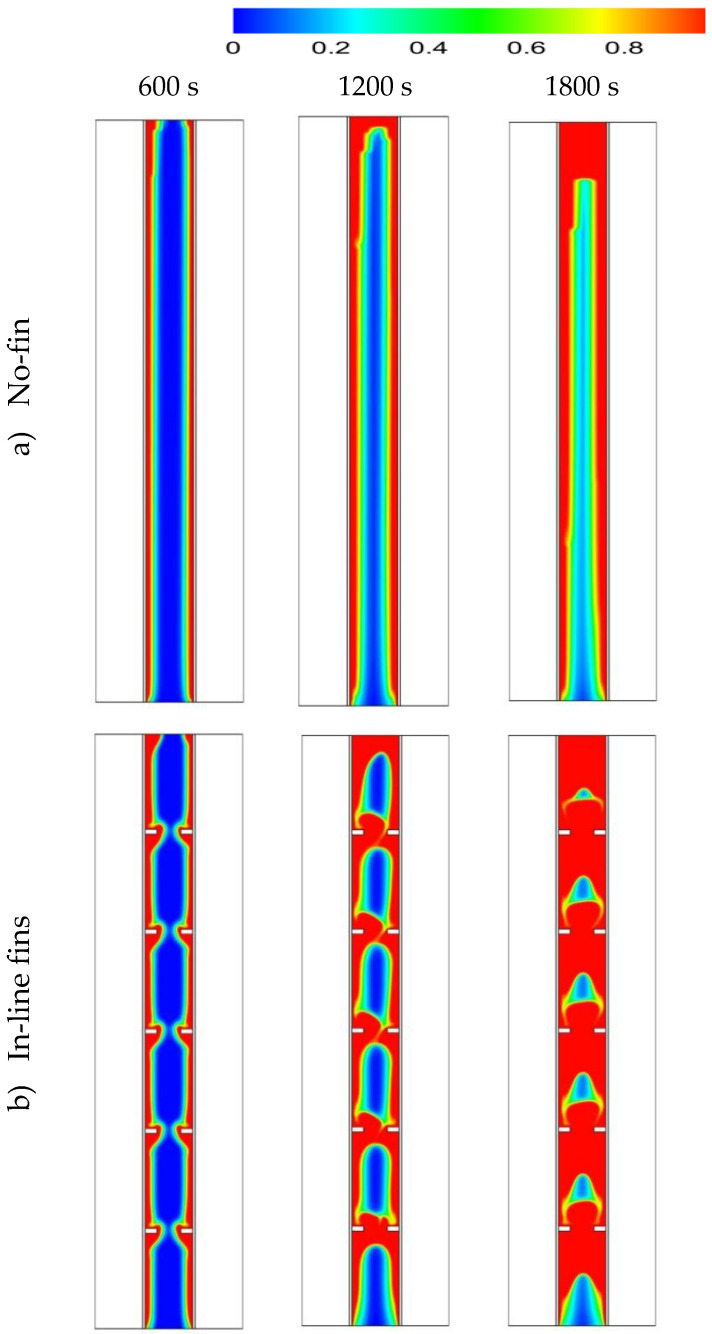

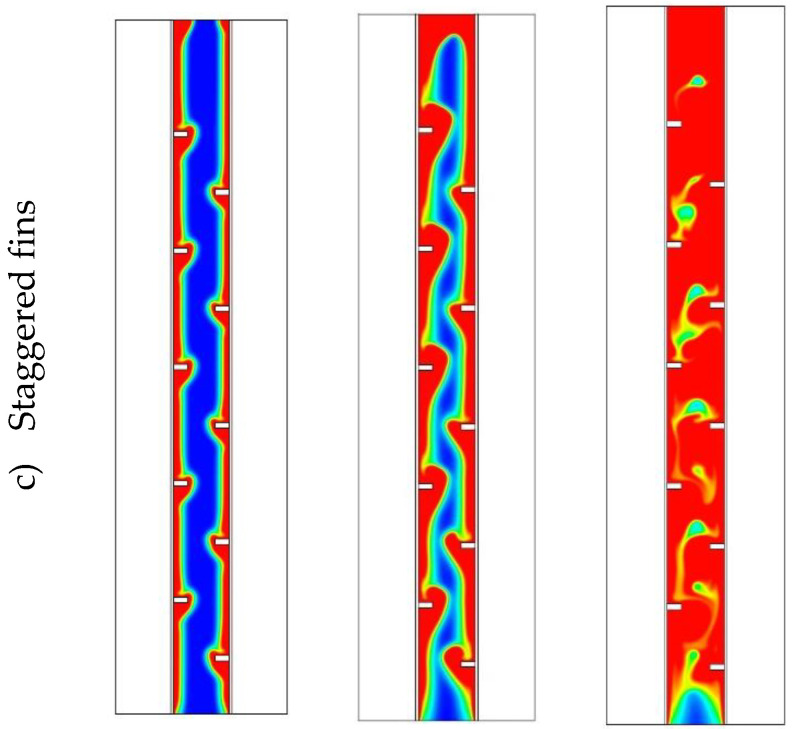
The liquid fraction development during 1800 s in three time-steps for the cases of (**a**) no fins, (**b**) inline fins and (**c**) staggered fins.

Figure 6 shows the liquid fraction development in the cases of no fins, inline fins and staggered fins for three different time-steps (600 s, 1200 s and 1800 s). In the case of no fins, the PCM melted in the area adjacent to the HTF walls first. Over time, more PCM melted, forming a thicker molten layer at the edge and agglomeration at the top (due to the natural convection), while the material at the bottom of the domain was still solid. At 1800 s, only 67% of the PCM had converted to the liquid phase and this value will improve using fins in the domain. Images in the second row of the figure show the inline-fin case. The two neighbor fins are separated by a distance of 40 mm, with a 40 mm separation distance from the top and the bottom sides. Five fins combined to the outer wall and five to the inner wall. The PCM begun melting at the region beside the wall and around the fins. Over time, the liquid fraction expanded to cover a larger area, the solid part divided in several portions by every two opposite fins. The PCM between the opposite fins melted entirely due to the release of more heat from the large surface area of the fins. By 1800 s, the total PCM melted was 90% and the remaining solid part was divided into 6 portions. Due to the natural convection, the liquid phase tried to collect at the top region of the domain. Therefore, the higher the location of the solid part, the smaller the solid size detected. This means that the larger solid phase in the domain is still at the bottom. To solve this issue, the staggered fins pattern was supposed (as shown in the third row of Figure 6). The first fin on the outer wall was separated from the bottom by a distance of 20 mm to release more heat to the bottom side, the upper fin was separated from the top side by a distance of 60 mm and the space between the fins was still 40 mm; however, the fins attached to the inner wall were distributed as the previous case. The solid PCM shrank between the fins in a continuous pattern and serpentine shape. In this case, the heat distributed relatively better than the inline case, especially at the bottom region, due to the lower number of fins in that area. A total of 92% of the total PCM melted within 1800 s and the remaining solid phase was distributed at different elevations, with the larger portion at the base.

**Figure 7:**
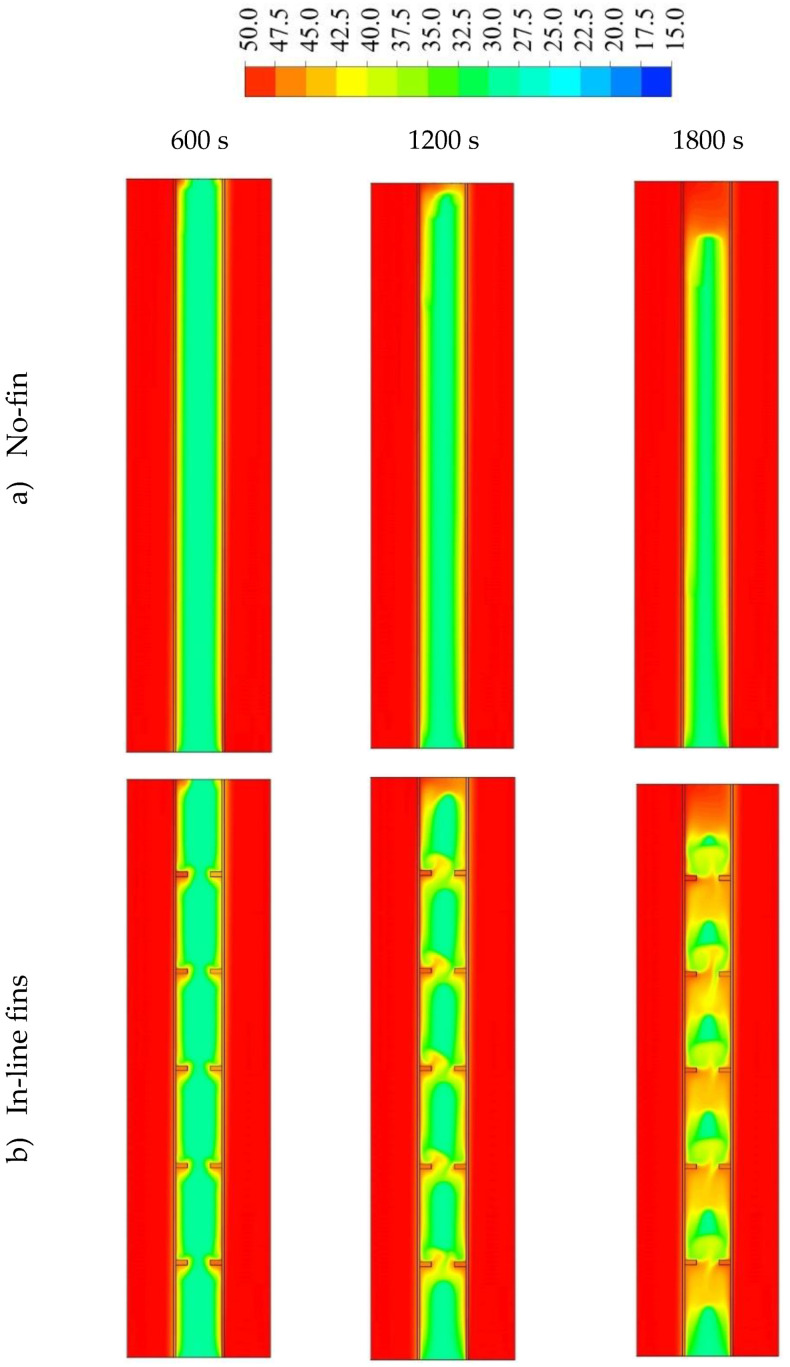

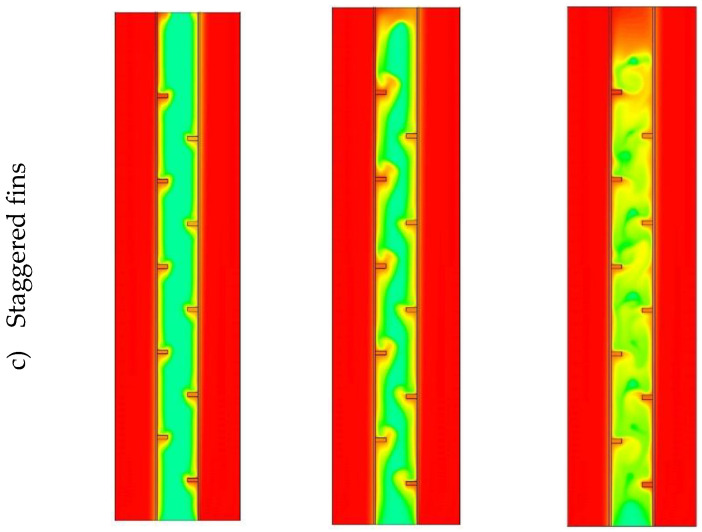
The temperature distribution (in °C) during 1800 s in three time-steps for the cases of (**a**) no fins, (**b**) inline fins and (**c**) staggered fins.

Figure 7 shows the temperature distribution during the 1800 s for the cases of no fins, inline fins and staggered fins. The zones of the heat transfer fluid remained at a constant temperature (shown in red color) for the period of the melting process because of the short length of the channel. For the case with no fins, the temperature rose by the line adjacent to the HTF channel walls. This temperature reduced toward to the center of the PCM domain. Due to the natural convection, the liquid-generated PCM gathered at the top part, which explains the high-temperature region at the top part of the domain. The temperature of the top region reached the equilibrium state with the HTF and, over time, the equilibrium region expanded through the domain. Within 1800 s, only 12% of the PCM achieved a balanced state with the HTF temperature. Because of the small size (2 mm × 5 mm) of the applied fins, the temperature of the fins was virtually equal to the temperature of HTF. As mentioned, the fins caused more heat to pass to the PCM, in a uniform distribution. Although the small size of the PCM region had the same temperature as the HTF in the case with no fins, the average temperature in the case of inline fins was relatively higher. From the third row of Figure 7, by reducing the distance between the first fin (attached to the outer wall of HTF channel) and the bottom, in the case of staggered fins, the temperature distributed more uniformly in the PCM domain than in the other cases and the average temperature was higher.

**Figure 8:**
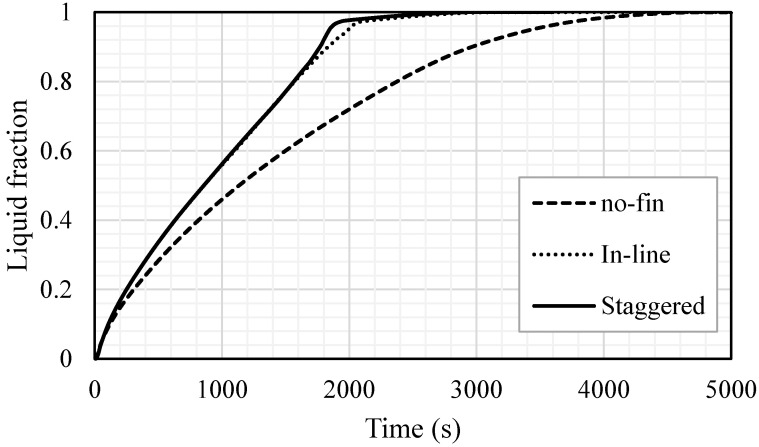
The liquid fraction development for all the PCM for the cases of no fins, inline fins and staggered fins in 5000 s.

In the case of no fins, the liquid fraction increased exponentially during the melting process, as shown in Figure 8. A total of 98% of the PCM melted within 4000 s and the whole PCM melted in 4727 s. The melting process decelerates over time due to the domination of the convection heat transfer over the conduction process. Within the inline fins, the charging process was achieved almost linearly in 2200 s, during which 96% of the PCM melted; then, it took around more than 800 s for the whole PCM to melt. Fast melting is caused by the fins, which deliver more heat through the system. The staggered fins enhance the melting process. The melting process followed the same procedure as that in the case of the inline fins within 1600 s; then, the melting rate accelerated due to the fins’ distribution and the location of a single fin close to the bottom.

**Figure 9:**
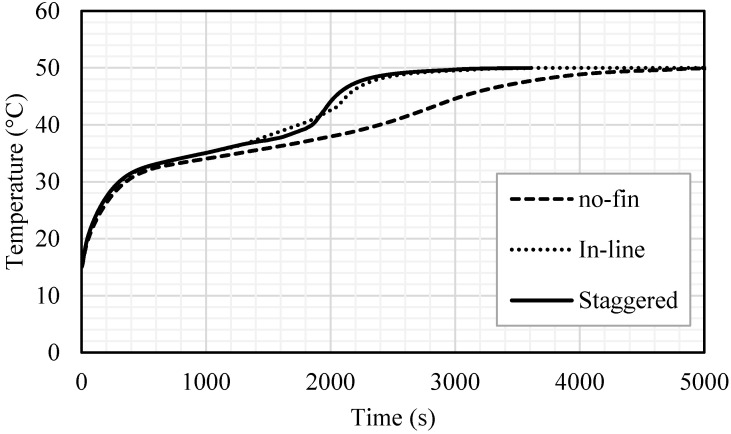
Temperature distribution during the melting process of the whole PCM for the cases of no fins, inline fins and staggered fins.

The temperature distribution during the melting process for all the cases is shown in Figure 9. The temperature in the no-fin case reached the thermal equilibrium with the HTF temperature very slowly (5000 s). It should be noted that, for the PCM, the time necessary to reach the thermal equilibrium is longer than the total melting time because, usually, the critical phase-change temperature is lower than the HTF’s critical point temperature. For the cases of inline and staggered fins, the PCM reached the thermal equilibrium faster (before 3000 s). It is worthy to know that the sharper alignment of the last two cases within 1800–2400 s happened because, by that time, most of the PCM converted to liquid; therefore, the heat transferred to the PCM was used to increase the temperature rather than to change the phase. Then, the slope of the lines becomes softer because the temperature difference between the PCM and the HTF was very small.

Table 1 shows the melting rate and power (heat charging rate) of the domain for the three mentioned cases. Using the inline fins and staggered fins accelerated the molten process by 33.7% and 37.2%, respectively. The melting power increased by 50.4% and 59.1% when the inline and staggered fins were used, compared with the case with no fins. The summary in this section indicates that using fins increases the efficiency of the system by accelerating the melting process and increasing the charging rate. Due to the location of the fins—close to the base of the PCM domain—staggered fins have a higher impact on the increase in efficiency than inline fins.

### 5.2. Effect of Size of the Fins on the Staggered Distribution Form of the Fins

**Figure 10:**
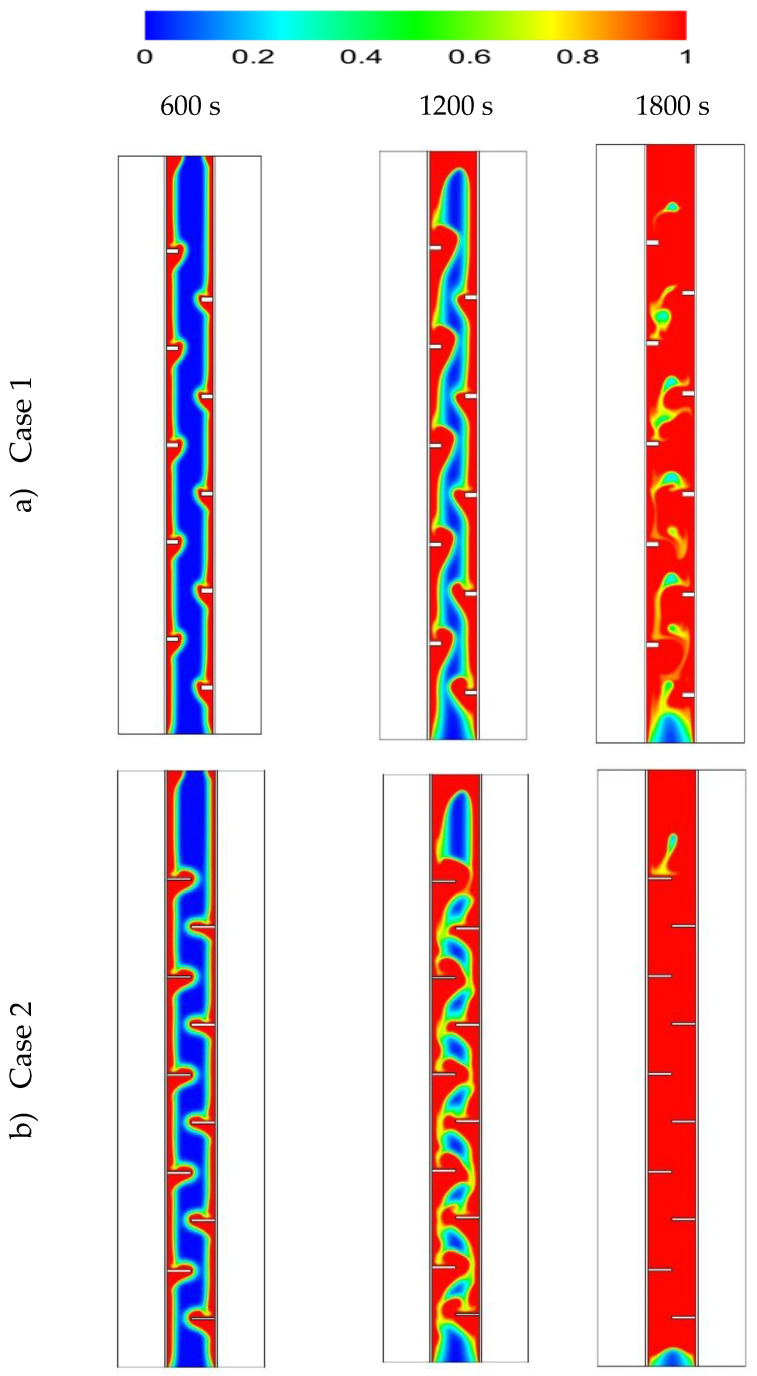

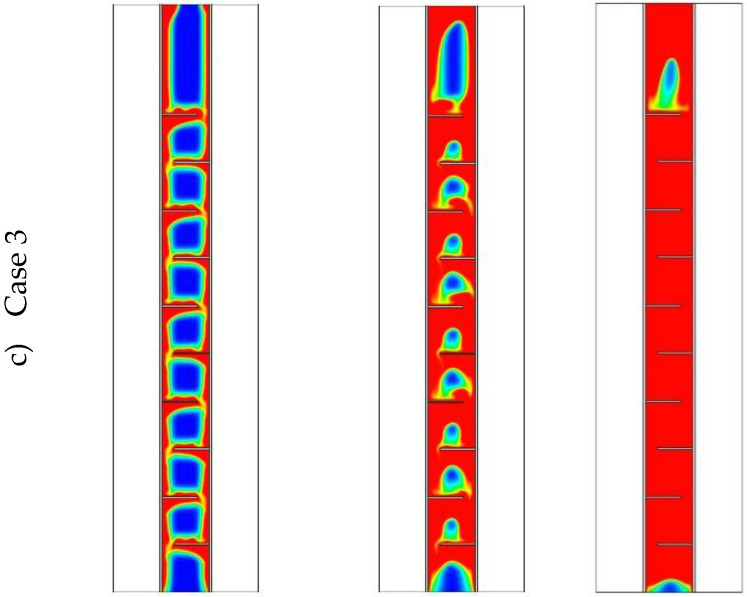
The liquid fraction development during 1800 s in three time-steps for different dimensions of the fins as (**a**) case 1 (2 × 5 mm^2^), (**b**) case 2 (1 × 10 mm^2^) and (**c**) case 3 (0.666 × 15 mm^2^).

The impact of the fins’ dimensions was examined considering the best case among the studied cases regarding the melting time and melting power, which was the staggered fin distribution. The dimensions studied include case 1 (2 × 5 mm^2^), case 2 (1 × 10 mm^2^) and case 3 (0.666 × 15 mm^2^) (W × L). Due to the considerable surface area of the longer fins with low thickness, the heat transfer rate to the PCM was higher, causing a faster melting process, as illustrated in Figure 10. However, the longer fins (0.666 × 15 mm^2^) reduced the open area (the gap between the fins’ edge and the opposite wall) generating a barrier to the flowing of the molten PCM due to natural convection and causing lower heat transfer by the natural convection effect. With short fins (case 1), the solid phase appeared as a serpentine; over time, the continuous solid state disappeared and produced a scattered, small portion of the solid PCM. Within 1800 s, 97% of the PCM had melted, in the case of the short fins. For case 2 (1 × 10 mm^2^), the melting process of the PCM approximately exhibited the same behavior as in the case of the short fins. In case 3, the melting process during the 1800 s achieved 97.5%, which shows an insignificant difference from the other two cases. The only different phenomenon that could be noticed is the restriction of the PCM between two neighbor fins. This phenomenon limits the ability of thermal expansion in the PCM domain.

**Figure 11:**
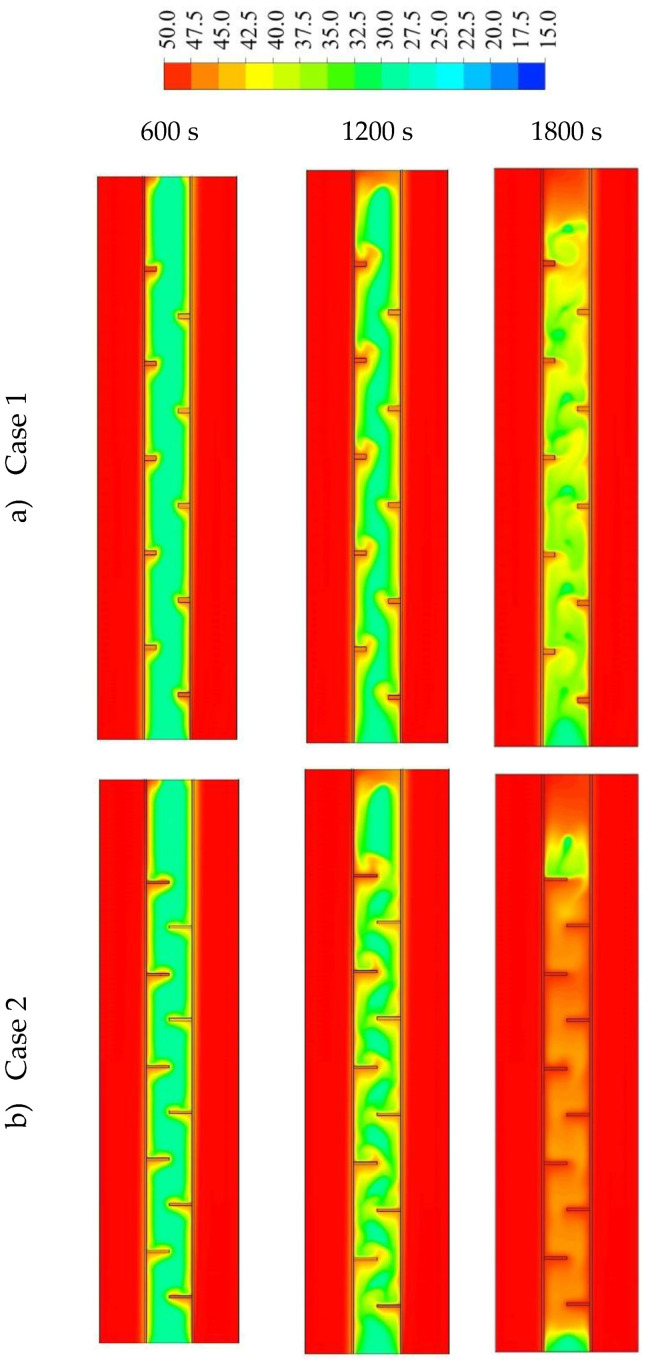

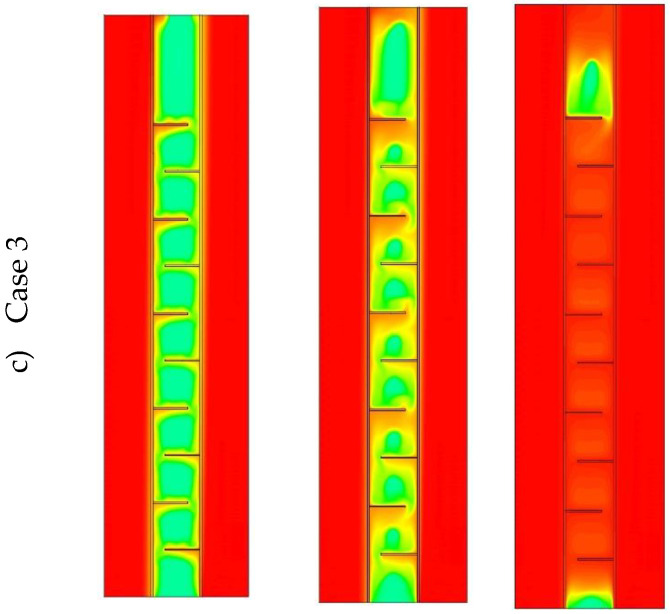
The temperature distribution (in °C) during a melting duration of 1800 s in three time-steps for different dimensions of the fins including (**a**) case 1 (2 × 5 mm^2^), (**b**) case 2 (1 × 10 mm^2^) and (**c**) case 3 (0.666 × 15 mm^2^).

Figure 11 shows the temperature distribution in the PCM domain during the 1800 s for all the cases. With the short fins (case 1), the temperature distributed uniformly but in slow steps. The warmer region gathered at the top and the cold portions collected at the bottom; this is because, in the case of short fins, the liquid phase had wider free space to move due to the natural convection. Within 1800 s, the average temperature reached 39.5 °C in the whole domain. Using longer fins (case 2: 1 × 10 mm^2^) helped the temperature to rise over all the domains, due to the large surface area of the heat transfer. In this case, the liquid phase had relatively enough space to circulate in the domain due to natural convection. The average temperature in the domain reached 47 °C in 1800 s. For case 3 (0.666 × 15 mm^2^), as mentioned previously, the PCM was restricted between the two neighbor fins with a narrow gap between the fin and the opposite wall. The heat transferred faster due to the large surface area of the fins; however, the length of the fins limited the circulation through the entire domain. On the other hand, circulation generated in the area between the two neighbor fins. The average temperature in the entire domain was 47.2 °C. The areas between the fins almost reached the thermal equilibrium with the HTF temperature. However, for the upper part, the PCM received heat from the lower fin and the upper fins were missing because of the wall, which caused a solid portion to remain at the top section of the domain.

**Figure 12:**
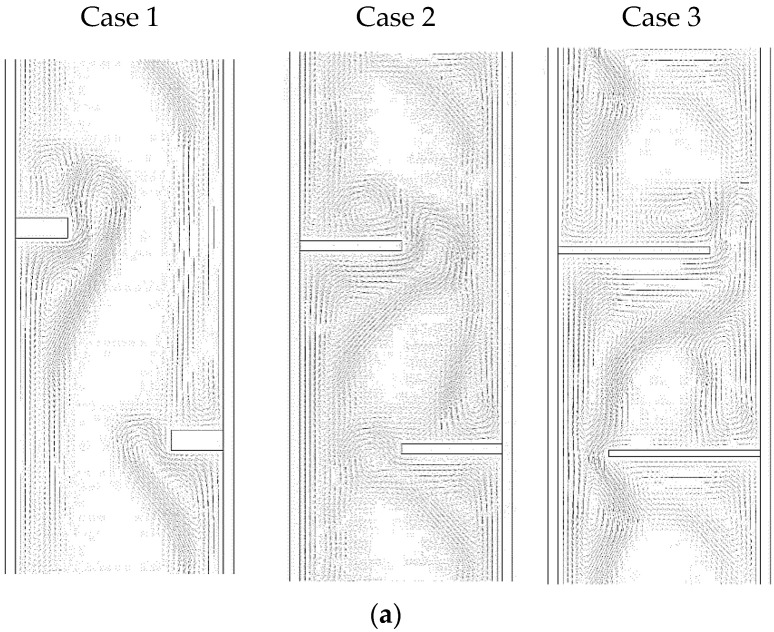

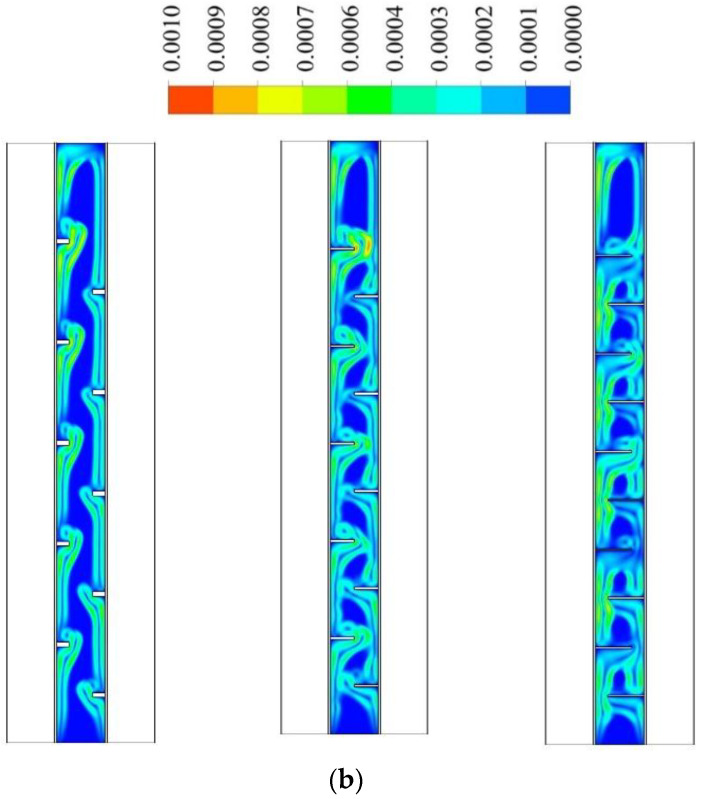
Streamline of the liquid PCM for the different lengths of the fins for the staggered arrangement ((**a**) vector streamline and (**b**) contours of the flow).

The circulation of the liquid PCM for the three cases at the time of 1800 s is illustrated in Figure 12a. The figure shows that the stream flowed in an anticlockwise direction through all the domains when short fins were used. In addition, there were small vortexes beside each fin because the fins formed a barrier for the streamflow. By using fins of 1 × 10 mm^2^, the flow showed different patterns but still created two vortexes, one through the whole domain and the other beside each fin. The longest fins (0.666 × 15 mm^2^) caused the main circulation to appear in the space between two fins. There were also small vortexes in the area far from the flow line, especially at the angle between the fins and the wall. The stream of the flow for the three cases is illustrated in the color mode in Figure 12b. The blue color presents either the area of the solid state or the stationary PCM liquid. The figure clearly shows that the flow was generated in the area where the heat is penetrated in the PCM domain.

**Figure 13:**
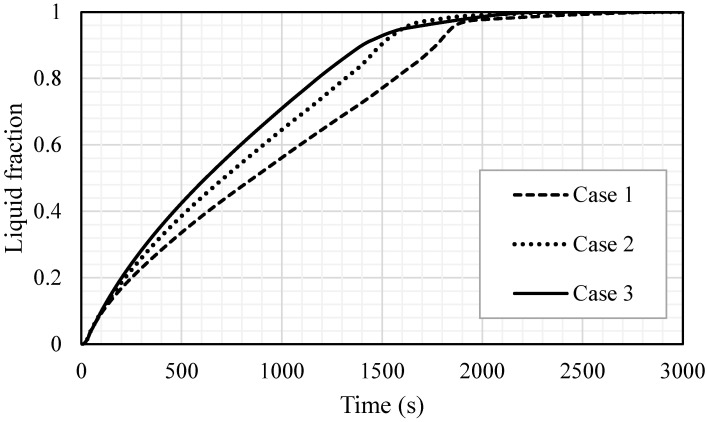
The liquid fraction development for the total PCM for the cases (case 1 (2 × 5 mm^2^), case 2 (1 × 10 mm^2^) and case 3 (0.666 × 15 mm^2^)).

**Figure 14:**
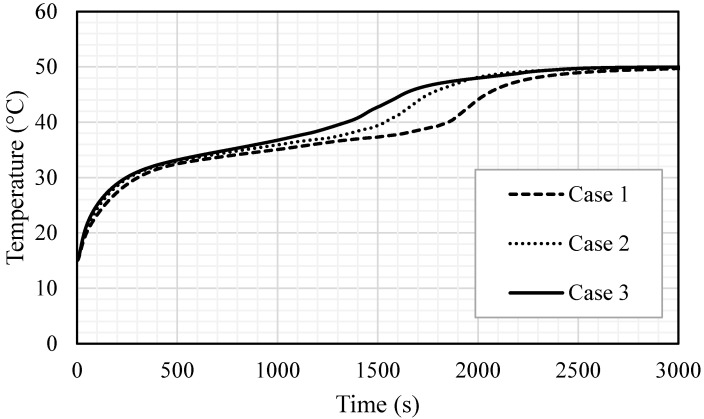
The temperature profiles for the total PCM for the cases (case 1 (2 × 5 mm^2^), case 2 (1 × 10 mm^2^) and case 3 (0.666 × 15 mm^2^)).

Figure 13 shows the liquid fraction development for the three cases in 3000 s. For case 1, the liquid fraction developed at a constant rate up to 1800 s; then, due to the natural convection effect, the heat transfer rate diminished and total melting was achieved within 2965 s. The curves for the other two cases (2 and 3) show a constant development rate of the liquid fraction up to 1550 s; then, the liquid generation rate diminished and the total melting times were 2557 s and 2396 s for cases 2 and 3, respectively. The average temperature increased significantly by the first 200 s due to the conduction effect, as shown in Figure 14. The generation of the melting process adjacent to the wall and the developing of natural convection, causing a rising temperature rate, diminished for all the cases. Case 3 showed a higher value of temperature and faster rising due to the high surface area of the fins. Table 2 shows that the melting time were reduced by 13.8% and 19.2% when the fin lengths were 10 and 15 mm, compared to the case of 5 mm. The melting power increased by 16% and 23.6%, when the fins lengths were 10 and 15 mm respectively, compared with the case of 5 mm. The summary of this section indicates that the longer fin presents a higher surface area of heat transfer, which means a faster melting process and higher charging power. Consequently, the efficiency of the system obviously improves.

### 5.3. Effect of Reynolds Number (For the Best Case—Case3)

**Figure 15:**
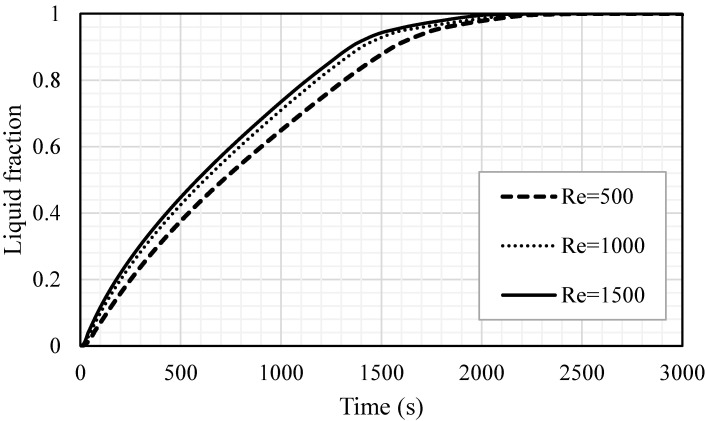
The liquid fraction development for the whole PCM for various values of Re (Re = 500, Re = 1000 and Re = 1500).

**Figure 16:**
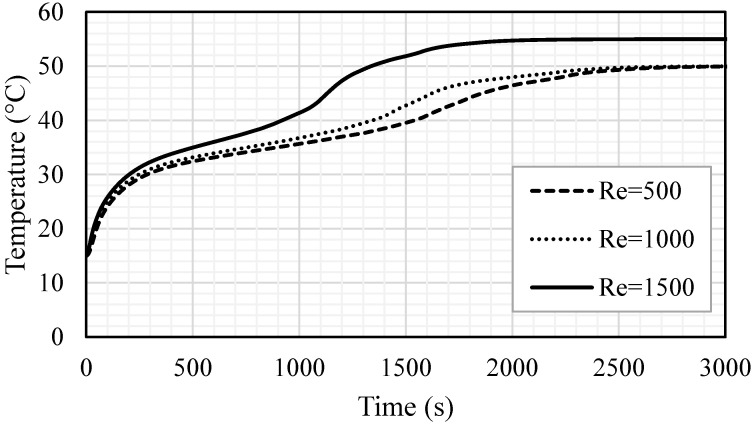
The mean temperature for the whole PCM for various values of Re (Re = 500, Re = 1000 and Re = 1500).

The effect of the heat transfer fluid velocity (represented by Re) was examined for the different Reynolds numbers of 500, 1000 and 1500, as showed in Figure 15. The velocity magnitudes related to the Reynolds number of 500, 1000 and 1500 were 0.01345, 0.0269 and 0.04035, respectively. A higher flow rate often improves the heat transfer ability and the convection process value. The considered Re was in the laminar flow range and the effect of the Re on the melting process was insignificant. For various values of Re, the melting procedure, gradients and melting rate were approximately similar. The time for the total melting was found to be 2544 s, 2396 s and 2310 s for the cases of Re equal to 500, 1000 and 1500, respectively. In the temperature profile study (shown in Figure 16), the mean temperature profiles for the case of an Re of 1500 show an advantageous behavior, compared with the other cases. In 2200 s of operation time, the PCM reached a stable value of 55 °C, while, for the cases of Re equal t 500 and 1000, the average temperature of the PCM reached a stable value (50 °C) within 2500 s and 2700 s, respectively. The change in the gradient, shown for the highest Re, started at 1100 s and was caused by the melting process, which developed a thermal convection effect. Even the differences in the system performance are insignificant, when using different values of Re, but the small improvement regarding one parameter should be considered to improve the performance of the entire system. Table 3 shows that the increase in Re from 500 to 1000 and 1500 reduced the melting time by 5.8% and 9.2% and improved the melting power by 6.2% and 10.3%, respectively. In summary, a higher Re improves the performance of the system by reducing the melting time and increasing the melting power.

Table 4 presents the pressure drop between the inlet and outlet pipes of the heat exchanger for different Reynolds number. The pressure drop increased with the increase in the velocity of the fluid in the pipes due to the higher friction loss in the pipe. However, as presented, the difference between the values of pressure drop for different Reynolds number is negligible; thus, a higher Reynolds number is preferable due to having a higher melting rate.

### 5.4. Effect of Inlet Temperature of HTF

**Figure 17:**
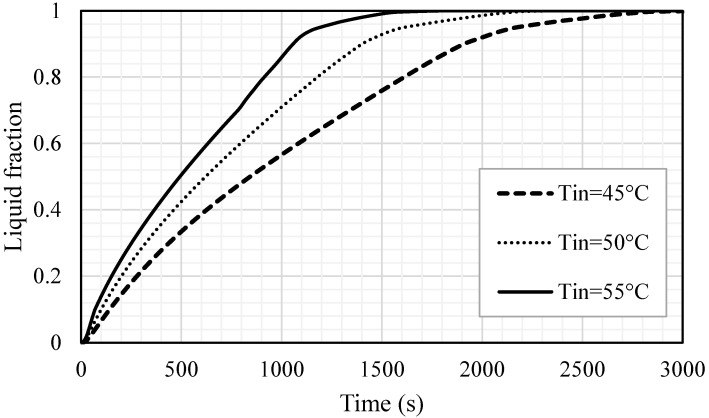
Liquid fraction development for the whole PCM for different cases of HTF inlet temperatures (Tin = 45 °C, Tin = 50 °C and Tin = 55 °C).

**Figure 18:**
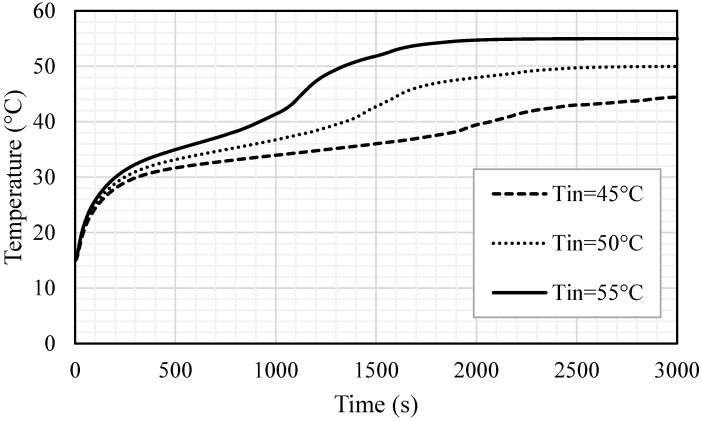
Mean temperature for the whole PCM for different cases of HTF inlet temperatures (Tin = 45 °C, Tin = 50 °C and Tin = 55 °C).

The impacts of the inlet temperature on the melting process are also here examined for the optimum case. Three different temperatures, 45, 50, 55 ℃, were used for this analysis. Figure 17 shows that the liquid fraction is significantly influenced by the change in the inlet temperature of the HTF and the melting time is inversely proportional to the inlet temperature. A higher inlet temperature caused a great temperature difference between the bulk on the wall; consequently, more heat transfer from the HTF to the PCM resulted in a faster charging process. The melting time diminished by 783 s and 1342 s, when the inlet temperature increased from 45 °C to 50 °C and 55 °C, respectively. The inlet temperature of the HTF expectedly reached the mean temperature of the PCM and the PCM reached a higher temperature utilizing the warmer HTF, achieving the thermal equilibrium with the HTF sooner. When the inlet temperature was 55 ℃, the PCM achieved the thermal equilibrium within 2000 s; however, the time increased to 2500 s and 3000 s when the inlet temperatures were 50 and 45 ℃, respectively (shown in Figure 18). This behavior is caused by a higher temperature difference, which causes more thermal exchange between the HTF and PCM and results in reaching the thermal equilibrium faster, in the case of the highest HTF temperature. Table 4 demonstrates the impacts of the inlet HTF temperature on the melting process. The total melting time was reduced by 42.2% and 24.6% when the inlet temperatures used were 55 °C and 50 °C, respectively, compared with the melting time when the inlet temperature was 45 °C, which is 3179 s. The charging power also improved by 88.4% and 38.5%, when the inlet temperatures were 55 °C and 50 °C, respectively, compared with the melting power when the inlet temperature was 45 °C, which is 50.7 W.

## 6. Conclusions

Circular fins with staggered distribution are proposed in this study to boost better thermal response rates of PCMs in a vertical triple-tube heat exchanger (TTHE) involving two opposing inflow streams for the heat transfer fluid (HTF). A set of simulation studies for PCM melting in the TTHE system with different fin configurations, fin dimensions as well as different HTF flow boundary conditions were conducted and reported. The potential of these parameters on modifying the melting enhancement rates of PCM was evaluated in terms of local temperature distribution, liquid fraction evolution, streamline distribution, melting time and charging power. Based on the existing results and their discussions, the following conclusions can be drawn:

(1) The inclusion of circular fins using inline and staggered arrangements reduces melting time, improving the PCM’s potential for better melting and higher heat charging rates. The results show that the melting rate and heat charging rate can be increased by 33.7 and 50.4%, respectively, in the case of inline distribution, and by 37.2 and 59.1%, respectively, in the case of staggered distribution.
(2) It is recommended to use longer fins and decrease fin thickness for the same fin volume usage to improve the potential of circular fins of staggered arrangement for melting enhancement in PCM-based storage systems. When the fin dimensions were (1 mm × 10 mm) and (0.666 mm × 15 mm), respectively, the melting rate was found to be increased by 16 and 23.6%, respectively, when compared to the base case of 2 mm × 5 mm.
(3) When applying circular fins in a staggered arrangement in a triple-tube storage system, the flow Reynolds number and inlet temperature of the HTF play a significant role in melting enhancement. The results confirm that increasing the Reynolds number from 500 to 1000 and 1500 decreased the melting time by 5.8 and 9.2%, respectively. Meanwhile, increasing the inlet temperature from 45 to 55 and 50 °C reduced total melting time by 42.2 and 24.6%, respectively.

It is worthy to mention that the idea of using circular fins with inline and staggered arrangements in PCM-based triple-tube systems with two opposing HTF flow streams has not yet been experimentally tested. Numerical predictions from this study reveal an encouraging improvement in storage performance when these techniques are used. However, experimental validation of their applicability would be more useful for changing and improving the design and operation of these systems. Furthermore, some additional ideas can be recommended to be implemented in the future, including studying the effects of the pressure drop of the HTF, utilizing a hybrid nanofluid as a HTF to improve the performance of the thermal efficiency and developing a more rigorous method for the configuration and distribution of the circular fins.

## Figures and Tables

**Table 1:** Melting time and melting power for the PCM for the cases of no fins, inline fins and staggered fins.

	Melting Time (s)	Melting Power (W)
No fins	4727	35.7
Inline fins	3136	53.7
Staggered fins	2965	56.8

**Table 2:** Melting time and melting power for the PCM for the cases (case 1 (2 × 5 mm^2^), case 2 (1 × 10 mm^2^) and case 3 (0.666 × 15 mm^2^)).

	Melting Time (s)	Melting Power (W)
Case 1	2965	56.8
Case 2	2557	65.9
Case 3	2396	70.2

**Table 3:** Melting time and melting power for the PCM for various values of Re (Re =500, Re = 1000 and Re = 1500).

	Melting Time (s)	Melting Rate (W)	Pressure Drop (Pa)
Re = 500	2544	66.1	175.13
Re = 1000	2396	70.2	175.32
Re = 1500	2310	72.9	175.55

**Table 4:** Melting time and melting power for the whole PCM for different cases of HTF inlet temperatures (Tin = 45 °C, Tin = 50 °C and Tin = 55 °C).

	Melting Time (s)	Melting Rate (W)
Tin = 45 °C	3179	50.7
Tin = 50 °C	2396	70.2
Tin = 55 °C	1837	95.5

## Data Availability

The data will be available on request.
